# Foreign

**Published:** 1840-09-19

**Authors:** 


### FOREIGN.

*Appendix to a former paper on Anomalous Affections of the Respiratory Organs.* By John Gairdner, M. D. F. R. C. S. Ed. (Read before the Medico-Chirurgical Society of Edinburgh, 14th April, 1840.)—In the end of the year 1833, I presented to this society a few cases of anomalous affections of the respiratory organs, which were afterwards published in the Edinburgh Medical and Surgical Journal, (Vol. xliii. p. 257.) In that paper I pointed out, that there are various convulsive actions of the respiratory muscles, which, although in some respects dissimilar, have such a general resemblance in others, as to justify us in classifying them together, and in suspecting some common origin. One instance of this sort is the incessant hiccup with which all who have had much experience in the treatment of diseases are familiar. I produced a case of pertinacious sneezing, supplied to me by my friend Dr. Beilby, and a case of convulsive sonorous expirations, resembling howling or screaming rather than coughing, of which a patient of my own was the subject. The details of these will be found in the 260th, 261st, and 262d pages of the volume referred to. These affections, whether of hiccuping, sneezing, or screaming, agree in the following points. 1. They are all of them convulsive actions of the respiratory muscles, consisting of a sudden sonorous expiration, repeated at short intervals; not of a rapid succession of such expirations, as in fits of common coughing. 2. They harass the patient, either without any interval, or with very short intervals, during his waking hours. 3. They are entirely suspended during sleep. 4. They generally continue for several days, sometimes for weeks, and are very liable to recur in those who have once had them.

Since the date of the above-mentioned communication, a relation of mine in coming from London by sea, had on board of the steamer with him a lady, who was subject to this sort of howling or screaming. It went on incessantly, and from the description my friend gave of it, I can have no doubt of the identity of the disease with the case just mentioned. Though a rare disease, it is therefore probably to be met with occasionally; and any suggestions as to the method of treating it will therefore, I trust, appear of some interest to a society of medical practitioners. It is in this point of view that I beg to offer the following case.

The subject of it is a lady, who might perhaps be approaching her thirtieth year, at the time when this narrative commences. She is of a remarkably mobile temperament, much more so than the subject of the former case. She was seized in September, 1836, with pretty severe headach, which was speedily succeeded by sonorous expirations at short intervals, going on incessantly. Each of these was performed with a sudden convulsive effort of the respiratory muscles, and with a remarkable jerk of the *recti abdominis.* Various anodyne and antispasmodic draughts, as well as enemas, were employed, composed of morphia, henbane, castor, saffron, and other like agents, alternated with purgatives. Scarcely any benefit was derived from any of these. The headach became accompanied by great intolerance of noises, so that very inconsiderable sounds startled her excessively, and roused the convulsive action. Some confusion of thought came on, and some acceleration of pulse, and I became apprehensive of the existence or approach of inflammation of the brain or of its meninges. I therefore cupped her twice on the head, as near as possible to the seat of her pain, drawing a large quantity of blood both times. She was greatly benefited by both bleedings, and the complaint subsided early in October, after having been present about a fortnight. I may remark, that though she had sonorous expirations during this attack, they were more like the exclamations one gives on being suddenly startled, than like the shrill scream or howl of the former case.

Early in November an alarming sound in the street revived the starting, which yielded, however, to an opiate followed by a purgative.

About three weeks after this, the old symptoms came on, not, indeed, so bad as at the first, but with such severity, that I thought it prudent again to cup her on the head to the extent of twelve ounces of blood. This was attended with great benefit. She recovered in a few days, and went through the epidemic influenza in the January following, and the measles in the succeeding month of April, without any return of the symptoms above described.

In the middle of February, 1838, about the termination of the menstrual period, she had a severe headach, and, learning at this time some disagreeable particulars of the illness of an intimate friend, she suddenly gave an involuntary scream or howl, accompanied at the same instant by a convulsive rotation of the head with a sort of jerk towards one shoulder. The whole of this action was repeated every two or three minutes during all the waking hours, and was suspended during sleep. The sound was much louder than that which accompanied the startings in 1836. There was neither fever nor confusion of thought, and the complaint yielded in a few days to the remedies employed, which were chiefly cathartic. On comparing this lady's case with that of the other lady, formerly read to this Society, there appears to me to be a very close resemblance. In both the sound was intensely shrill and startling. Of the two the shrillness and suddenness were perhaps more remarkable in the subject of the present narrative, but the intervals between the screams were longer than in the other case. In the course of the two years which have since elapsed, she has had some ten or twelve different attacks of it, each lasting at least two or three days, often a week or more. Sometimes the howl has gone on every minute or two during all her waking hours. Sometimes it has been suspended for several hours, and has begun again. The treatment has consisted chiefly in the alternation of cathartics with tonics. Removal to the country in the summer was at one time of great service as a prophylactic.

In the month of March, 1840, she had a very obstinate attack, which baffled all the modes of treatment I had formerly resorted to, and appeared to call for some new and more efficient remedial method. Recollecting that the nerves of the respiratory muscles are all of them connected with the upper part of the spinal cord, I applied *a blister to the nape of the neck*, and was delighted to find that the disease yielded immediately. It recurred, however, after the lapse of some days, and became as bad as ever. I then urged the repetition of the blister, but could not persuade her to apply it, as she was not satisfied that her relief on the previous occasion had really proceeded from it. After in vain trying opiate and antispasmodic injections both into the rectum and the vagina, I at length prevailed on her to submit again to the application of *the blister to the nape of the neck*, close to the occiput. It had scarcely begun to act, when the howl, which had persecuted her for several days, was at once removed. This was on the 23d of March. The blister was kept open for several days by the application of the *Ungt. Sabinæ*, and on inquiring for her more recently, (10th April,) I found her still continuing free from her complaint.

When I sat down to write out this account, I was not aware of any similar cases having ever been treated on the same principle; but I have since made a pretty extensive search into the records of our profession, and have been much gratified to find that the same or similar theoretical considerations had induced others to adopt the same treatment in cases presenting many points of analogy, and that it had been attended with the same success.

In the annals of Medicine, (Vol. vii. p. 351–358,) Dr. Scott of the Isle of Man has recorded a remarkable case of a young lady, whose disease he describes as "somewhat resembling hiccup; that is, the inspiration was imperfect and sonorous, but not so convulsive, though more frequent than what we observe in genuine singultus. It was perhaps more in sound like the panting which occurs after violent exercise, or like an aspirate pronunciation of the interjection, Ha!" This disease resembled that of my patient in some very important particulars,—in the general character of the sound emitted, so far as one can judge of it from a verbal description; in the absence of fever, and of all acute symptoms; in the absence of all indications of organic disease; in being very loud and incessant during the day; in being completely suspended during sleep; and in having resisted a great variety of remedial measures. It seems to have declined gradually about six months after its commencement. She had a second attack of it, however, about three years after the first, which, after also resisting many remedies, yielded at once to a *blister applied to the back of the neck*, so as to cover completely the cervical vertebræ. It is curious and interesting to remark, that various other blisters had been previously applied in this case—to the throat and to the breast, between the shoulders, to the insides of the thighs, and to the side—and that all of these failed to remove the complaint. Dr. Scott says he was induced to apply the blister to the part where it ultimately proved effectual, "by reflecting that the diaphragm, the organ probably chiefly affected in the disease, was supplied principally by the phrenic nerve, which is formed by the junction of the third and fourth cervical nerves, and *from having had experience of the efficacy of blisters to the same parts, in cases of singultus in typhus*, which had baffled other means."

I have the pleasure to acknowledge the confirmation which this practice has received from the experience of Dr. Shortt of this city, who has inserted in the 39th volume of the Edinburgh Medical and Surgical Journal, (page 305—313,) a valuable communication "on Hiccup, its causes and cure." Dr. Shortt thus describes his practice, and the rationale of it. "Soemmering recommends blistering between the shoulders; but the most powerful means, so far as I have had any opportunity of judging, appears to be that of *blistering the surface of the neck over the origin and course of the phrenic nerves*, for which purpose the blister is applied nearly round the neck to secure its position, and to cover fully the different parts concerned." Dr. Shortt relates four cases in which this practice was successfully applied. The first was a case of common cholera, in which hiccup occurred as a symptom. The second, which was soon after fatal, was a case of disease of the liver and kidney with fever; in the course of which the same phenomenon presented itself. In the third case the hiccup was the effect of a blow on the mastoid process. In all of these cases, dissimilar as they are from each other, and from those I have detailed above, the hiccup seems to have yielded to the remedy. The fourth case presents a much closer resemblance to the subject of my present paper, as the case was unconnected with organic disease or local injury; and, to use the words of the author, "resembled a combination of coughing and sneezing more than hiccup, as the violent effort, consisting of the irresistible and involuntary expulsion of air from the windpipe, was produced during expiration; but the loud sonorous sound of the former did not exist, and no irritation of the nasal nerves was at any time apparent; while during the attack, the lower jaw was invariably drawn upwards and forwards as in sneezing." The disease, of which the subject was a boy of ten years of age, seems to have occurred by fits, which were most severe in the morning, and lasted sometimes three hours without intermission. They were entirely suspended in sleep, and liable to be excited by mental emotions. The convulsive efforts succeeded each other sometimes at the rate of 330 in twenty minutes, (or more than once in four seconds,) producing extreme exhaustion. The remedies unavailingly employed were the following: emetics; purgatives; local stimulants and astringents to the throat; an alterative course of mercury; opium and hemlock externally to the neck, chest, and spine; narcotics of very various sorts, and quinine, internally; also the warm bath and garlic poultices to the epigastrium. *The disease at once yielded five hours after the application of a blister* in the manner described by the author.

Dr. Bright has two very interesting cases illustrative of this subject in the second volume of his "Reports of Medical Cases." The first of these, (Case 211, p. 457,) a young lady, had laboured for a fortnight under an incessant convulsive effort, "something between a hiccup and an attempt to vomit," loud enough to be heard at the house-door when she was up stairs. The remedies employed "tranquillized the whole in the course of the night." They were, *a blister to the nape of the neck;* cold to the forehead; frequent doses of sulphuric æther; and purgatives of cathartic extract, and the compound galbanum pill. I need not say that I attribute the prompt cure to the first of these.

The other case (Case 212, p. 457,) a girl of eighteen, had been for ten months subject to an involuntary sighing, much increased during the preceding six weeks. "She was unceasingly uttering a sound, like 'heigh-ho! heigh-ho!' at regular intervals of three seconds, so that the sound was repeated twenty times in every minute, unless it was at times changed for the single word 'heigh!' which was then repeated thirty times in the same period. This sound was, however, to a certain degree and for a short time under her control, so that she could check it, with much apparent exertion, for a few moments, while she uttered a short sentence; but it was immediately resumed when she ceased to speak; and if she attempted to put two or three sentences together, they were interrupted by this spasmodic and almost involuntary sound." The remedies employed were, a cold wash to the shaved head; *a blister between the shoulders;* colocynth with calomel, aided by senna; and a camphor mixture with æther every six hours. The spasms gradually subsided towards 4 o'clock of the following morning.

On comparing these two cases with that which I published in the forty-third volume of the Edinburgh Medical and Surgical Journal, (p. 260,) a great similarity will be apparent in the general character of the disease, though not in the speediness of the cures. I was not then aware of what I now deem the just principle of the treatment, and I certainly attribute Dr. Bright's greater success to the superiority of the methods he pursued. I only regret that he employed simultaneously such a variety of remedies, as the really efficient ones are thus deprived of a part of the evidence in their favour. I trust this evidence will appear more clearly by grouping Dr. Bright's cases, as I now do, with others of a similar description.

I have not been able to discover any other recorded cases which possess the precise characters enumerated at the beginning of this paper, though I doubt not that a more extensive search might detect many such; but there are many instances of convulsive coughing, unconnected with hooping-cough, or with organic disease, which are probably referrible to similar causes, and will in all probability yield to similar treatment. An excellent and well-detailed case of this sort will be found in the Edinburgh Medical and Surgical Journal (vol. vi. p. 499,) and many similar ones may be found in the writings of physicians. I shall content myself with giving one example of this sort of cough, because it possesses considerable interest in reference to the subject of this paper, on account of the remedies employed. It is to be found in the sixth volume of the Medical Commentaries, page 343, and is related by Dr. Charles Leith, at Johnstone, near Montrose.

The subject was a girl of fourteen, affected with a most violent convulsive cough, occurring by fits, with intervals of from half an hour to three hours, or even longer. The duration of the fits was from five minutes to twenty minutes at a time. Unlike some of the former cases, it had all the character of a genuine cough, or rather of a hooping-cough; for it seems to have been as violent as the very worst pertussis, and was alternated with very loud sonorous inspiration. She had passed through the real hooping-cough some years before. Dr. Leith does not say it was suspended by the supervention of sleep, but it had been several days in existence, when he was consulted.

The white oxyde of zinc had at that time a high reputation, and she was directed to take one grain every six hours. She had but one slight return of her cough on her way home from consulting the doctor, who attributes her cure to the oxyde exclusively, though he had also ordered her to be bled, and to have *a blister applied to her neck.* He accounts for his opinion, by stating that the cough had ceased before eitherof these remedies had been applied. This reason is tolerably valid as to the bleeding which was not performed till the following, day; but it is by no means equally valid as to the blister, which was applied the same evening. The first dose of the oxyde waht taken at four o'clock, and, as the cough had previously to that time been sometimes several hours suspended, it is strange that he should not have thought it possible that its permanent suspension might be the effect of the blister. To our modern apprehensions, a grain of white oxyde of zinc taken every six hours, seems a very inadequate cause of its final cessation. I am much disposed to think that the blister was the efficient remedy in this interesting case.

I am very sensible of the caution necessary in drawing general conclusions from so limited a number of facts. Yet it is impossible to avoid speculating, even if it were desirable to do so; and as I agree in an opinion ascribed to the celebrated Dr. Cullen, that false theories are far less injurious in medical science than *false facts*, I shall conclude this paper with a few inferences, to which the preceding details seem to me to point.

1. As the various actions above described, whether of coughing, hiccuping, sneezing, screaming, or a combination of two or more of these, involve the simultaneous movement of a variety of muscles connected with the respiratory apparatus, and as these muscles are supplied with nervous influence chiefly by the phrenic and pneumogastric nerves, which originate from what has been termed the respiratory tractus, so particularly described by Sir C. Bell, it seems highly probable that the power of the blisters over these affections depends on some influence exerted by them on the state of the upper part of the spinal cord; and this inference is corroborated by the fact, that in one of the cases above related, blisters applied in other situations were totally inefficient.

2. It does not seem to me to be established by the facts, or rendered probable by any theoretical considerations, that the application of the blisters round the greater part of the neck, so as to follow the course of the phrenic nerves, as recommended by Dr. Shortt, is essential to success. It is evidently a severe procedure. It is equally obvious that it is not always necessary; and it seems probable that the cure is in no degree owing to the application of the blisters over the course of the nerves; and that their benefits will be fully obtained if they are applied on that part of the spinal cord from which these nerves originate.

3. It seems to me probable, (though upon this subject I would be understood to speak with great diffidence,) that the pathological and therapeutic principles above indicated may, if well founded, be of more extensive applicability, than to the forms of disease which I have been endeavouring to describe; and that all spasmodic affections of the respiratory muscles, which are not excited by inflammation, or by organic changes in some part of the respiratory apparatus, nor by mechanical irritation of the windpipe, or of its subdivisions, may admit of alleviation by similar treatment. I would therefore suggest the propriety of trying the effect of the application of blisters to the upper part of the cervico-spinal region in two diseases of this description;—I mean hooping-cough and *laryngismus stridulus*, or the crowing disease of infants. Both of these produce violent spasmodic action of the respiratory muscles; and both are occasionally attended with great danger to life. Both, therefore, are cases which would warrant the application of a much more severe remedy, provided that it held out even a speculative prospect of alleviation or of cure. The researches of Watt and of others have sufficiently established that pertussis is often accompanied by inflammatory affections of the trachea and bronchi; but they have not established that those affections are the true causes of the violent cough, the very character of which leads us to attribute it to some irregular action of the nervous system. The very ingenious and beautiful theory of laryngismus, proposed by my much esteemed friend, the late Dr. Hugh Ley of London, may be the true explanation of some cases of that disease; but I have had a dissection since the promulgation of Dr. Ley's theory, which proves to me that it is not applicable to all cases of that description. The enlarged bronchial glands of Dr. Ley, pressing on the recurrents, are sometimes not to be found by the most careful dissection; and there seems, therefore, to be a necessity, in such cases at least, for referring the complaint to some cause influencing the respiratory nerves at their origin. This view of the pathology of this singular morbid affection is by no means peculiar to me, but has presented itself to several of those who have written upon it. In an excellent treatise published in 1830, by Dr. Marsh of Dublin, (Dublin Hospital Reports, Vol. v. page 616,) in which he treats of this disease under the title of "Spasm of the Glottis," the author, after establishing by dissection the absence of all cerebral changes adequate to explain its symptoms, says, "It would be interesting on any future occasion to examine accurately the state of the pneumogastric nerve. The seat of the disease may perhaps be found to exist at the origin of this nerve; and topical applications made as nearly as possible to its origin, may be found to constitute an important part of the treatment." In this opinion, or rather hypothesis, I exactly coincide; and I merely observe in addition, that of all "topical applications" it appears to me, on the strength of the analogies I have endeavoured to establish, that blisters afford the best chance of success, and are best deserving of a fair and impartial trial.

Dr. Bright, in the remarks with which he concludes his very interesting cases of hydrophobia (Reports &c. Vol. ii. pages 605—607,) proposes as remedies for that formidable disease, "cupping, leeches, moxa, and blisters about the nape of the neck, and the upper part of the spine;" and he has evidently been impelled to this proposal, by the consideration that the symptoms of hydrophobia "seem to be immediately dependent upon some morbid action excited in the nerves of organic life, or that particular set of nerves which has been so beautifully illustrated by Mr. Charles Bell as connected with the respiratory apparatus."

I might pursue these analogies to the theory and treatment of some forms of spasmodic asthma; but I shall not at present press that hypothetical conjectures any further. I trust I may have succeeded in making out a case of sufficient strength to induce my professional friends to make a trial of the practice; and as some of the forms of disease to which it is applicable are by no means of frequent occurrence, I hope they will be induced to favour the society and the profession with the results of their experience.

I have only to add, that I shall be greatly obliged by being made the channel of such communications, from those who may peruse what I have written on this interesting subject.—*Ed. Med. and Surg. Journ.*

---

*Mercurial Trembling.*—Francesco Boeri, aged fifty-five, an Italian looking-glass maker, of short stature and dark complexion, was admitted into the Charing-Cross Hospital, under the care of Dr. Chowne. He stated that he had worked at the trade in this country twenty-eight years. He had been a muleteer in his own country. During the first five years of his employment in the manufacture of looking-glasses he had enjoyed good health, but at the expiration of that time he began to tremble.

He consequently left his work for two months, and then became as strong as ever. He returned again to his employment, and kept strong for about six years, and then the trembling came on again. In each attack he trembled a good deal; the hands were always the parts affected, the right hand the most so; and this was more employed than the left in his work. In each attack he could walk well; his knees trembled slightly, but not to signify.

He could keep his hands still when he rested upon any thing firm; but the attempt to take any thing in his hands increased the trembling. He was excessively nervous, and if any one even looked at him he shook the more. Anxiety always increased the shaking. The tongue was affected, and the difficulty of speaking was augmented by similar causes. The head sometimes shook a little.

During the whole period, the sense of touch was unimpaired; the vision seemed rather gloomy, or obscure. He sometimes felt as though he were drunk; his thoughts used to wander. He left his work for about two months, and again became well.

The present attack, the third, has been like the former attacks in all essential particulars. The legs, however, have been more affected than they were before; but still not so much so as the hands. He walks sometimes as if he were tipsy, and has often been taken for a drunken man in the streets. The eyes have been less gloomy, the sight less obscure. The tongue is more affected in this attack than it has been in any of the previous ones. Sleep generally good; the memory has been much impaired, more than at present.

During the present attack he has frequently felt as though there would be alvine action, but before he could reach the water-closet was seized with sudden loss of power, and dropped to the ground; the bowels at the same moment acting involuntarily. The falling down was generally, but not always, preceded by the sensation that the bowels would act. He has sometimes fallen and got up again, without the bowels acting; but, generally, it has been otherwise.

If he were not found and assisted to get up, an hour or even two hours would elapse, before he could sufficiently recover himself to rise. Prior to coming into the hospital he fell two or three times a week, and had the involuntary action of the bowels.

He thinks he should not have been so bad this time if he could have left his work as he did before; but he had had domestic troubles, particularly the loss of several very near relatives, and this he thinks might have also aggravated the disease. Although he has never been salivated, his teeth have been often loose; three of his teeth in front are much decayed: his parents had good teeth, and he does not think that the defect in his own is hereditary.

Upon his admission into the hospital, the general functions were proceeding naturally; the intellects were pretty clear, but subject to great embarrassment from hurry, fright, anxiety, or other excitement. Vision clear; appetite natural. No febrile symptoms or pain; pulse regular, soft, full, and eighty-four. Skin natural; sleep pretty good, tranquil, and refreshing; feels generally weak, in the legs especially. The head, the hands, and the legs trembled; the head least, the hands most. The weakness in the knees very great; the tongue trembling; the speech slow and tremulous, even in the absence of excitement. In every part, the trembling and agitation were increased by hurry and anxiety.

During the first few days he had a tonic steel medicine; but as this appeared to induce constipation, it was discontinued, and a stimulant, aromatic mixture with rhubarb, substituted. A strong stimulating liniment was ordered to be rubbed over the limbs and vertebral column. Under this treatment he considered himself, and appeared to be improving for about three weeks. His diet was nutritious.

At this time tonics were resumed without the least benefit. Without any obvious cause, a greater degree of debility supervened, the shaking increased, and the whole aspect of the disease became unfavourable. He gradually sunk into a state of greater and greater weakness, for which cordials and wine were administered without effect. This change was not accompanied by any specific change in any organ or function, but appeared to consist of a general failing of the vital powers. The intellect appeared to be clear, and, though weakened, not affected by aberration, until the accession of actual coma, soon after which the patient died.

*After-Death Appearances.*—The body was examined twenty-four hours after death. The thoracic and abdominal viscera presented nothing unusual. The membranes of the brain were in the natural state. The cerebrum and cerebellum appeared to be healthy throughout, except that to the touch, and when cut into, they seemed to be rather more firm and dense than usual. The colour was not quite so white as natural, and approached towards a shade of dullish yellow. The spinal cord presented no appreciable change. The ventricles were natural, and contained the usual quantity of fluid. The vascular system of the brain healthy.

Dr. Chowne, in lecturing on this case, observed, that as the patient, on his admission into the hospital, was apparently free from organic disease; as the natural functions were, for the most part, proceeding regularly; as the whole of the symptoms seemed to be the result of the employment he had followed; and, as on previous occasions, removal from that employment had been followed by removal of the disease; he had, after finding that the tonic he had prescribed was not without its ill effect upon the bowels, very willingly discontinued it, and prescribed only such medicine as was necessary to insure regular action of the bowels. The appetite continued good, a sufficient quantity of nutritious food was taken and well digested, and the sleep was sound and tranquil. When, however, there did not appear to be the desired improvement in the trembling, tonics were again had recourse to, but without effect, and the patient gradually sunk.

The result of the post-mortem examination was satisfactory, inasmuch as it showed that there was not any hidden organic disease of either the thoracic or the abdominal viscera; and that what departure from the healthy state existed, was in that organ in which the nervous affection might be well believed to have originated; he remarked, however, that he spoke with a degree of hesitation as to actual change in the structure of the brain. He would venture only to say, that there appeared to be, so far as the sight and touch could distinguish, a slight increase of the hardness and a slight change of colour; but he reminded the students that these were somewhat difficult distinctions, when the departure from the natural appearances was but slight. He also reminded them that such appearances were not necessarily the consequence of disease.

With regard to the diagnosis, the agitation and trembling, under which the patient laboured, resembled, to a certain extent, the agitation of delirium tremens, and the tremulous motions of the hands of persons debilitated by drinking, in whom the disease had not extended so far as to produce delirium with the usual febrile excitement. The diagnosis, however, was not difficult. In tremors from mercury, there was, almost always, entire absence of the spectral and other illusions of the senses, as of hearing and of the touch. There was also, generally, the absence of the profuse clammy sweats, of the restless disposition to get out of bed and to ramble about; of the constant watching; of the peculiar restless expression of the eyes; of the rapid, voluble, noisy talking; and of the mental aberration.

It might be distinguished, also, from the trembling produced by excessive drinking, where delirium tremens had not yet supervened, although here the dissimilarity was not so great. The history formed an obvious indication, and the person affected by mercury was so generally aware of the disease, that he could seldom fail attributing it to its right cause. Tremulousness from drinking, however, was generally, in its early stages, temporary, occurred most commonly in the morning, and was frequently removed as soon as a fresh supply of stimulus had been swallowed; the tremulousness from mercury, on the contrary, was not an affair of a short period; was not especially worse in the morning; and was not removed, or even diminished, by a glass of spirits.

The disease bore a stronger resemblance to paralysis agitans than to the tremor which is the result of drinking, either in its mild or severe form; and without some circumspection on the part of the practitioner might be easily mistaken for it. Although, however, the general resemblances were strong, there were specific peculiarities by which they might be distinguished. In this, as in trembling from spirituous liquors, the history was a prominent feature. The attack was generally gradual, and, for the most part, unconnected with symptoms of ordinary cold, while paralysis agitans was frequently preceded by exposure to cold or to damp, and would come on somewhat suddenly; the symptoms of cold passing away, and the paralysis remaining. There would be a further diagnostic guide in the character of the trembling, and in the circumstances under which it became augmented or diminished. In paralysis agitans, supposing the lower extremity, for example, to be affected, it would be seen to tremble, at all times, as well when the limb rested on the ground, or was otherwise supported, as when it was not supported. The trembling from mercury, on the contrary, was increased excessively by nervous emotion, or by muscular effort; the patient, on taking any thing in his hand, found the trembling the greater as he increased the effort, and it approached nearer the object of which he wished to obtain possession. The trembling was said to have been so strong in some, that they had not been able to convey their food to their mouths, and they had fed like quadrupeds. In some cases, as in that under consideration, the head was also affected with the trembling motion. In ordinary paralysis agitans, it often happened that the greater the effort required to perform any movement, the more steadily it was performed, and persons who could not walk without extreme agitation and irregularity, were able to run with tolerable steadiness. The progressive movements, therefore, of a person affected with paralysis agitans, were not unfrequently hurried and quick. This had not been the case with Boeri; on the contrary, increased anxiety and effort, instead of quickening his speed, would have brought him to the ground. The affection of the tongue was also greatly aggravated by efforts to speak, either faster or plainer than usual: when the mind became excited, and the endeavours to speak were increased, the power of articulation was almost lost. Thus we saw that the trembling from mercury appeared to agree in character with the tremors from excessive drinking, in being increased by muscular efforts, and in being greatly diminished, if not wholly arrested, by support; but that it differs in both these peculiarities from paralysis agitans.

During the whole time that Boeri had been in the hospital, his mental faculties had been perfect, with the exception of his liability to be confused by hurry, anxiety, or excitement.

With regard to the special circumstances which gave the predisposition to this disease, we knew nothing with certainty, beyond the fact, that some individuals were more, others less susceptible, and that the susceptibility varied in the same person at different times.

We found in the present case that the same individual worked with impunity five years, and kept in very good health; that on a second occasion he worked six years without suffering, although the first and the second periods had an interval of only two months. He next continued working with impunity fifteen years, at the termination of which time the present attack came upon him, and continued for two years, until his death. We saw also, in this case, the efficacy of abandoning for a time the occupation, or, in other words, the withdrawal from the exciting cause: in each of the former attacks a two-months' absence from the source of mischief was followed by recovery, and in this last attack he could not absent himself from his employment, and the disease continued. His own impression was, that if he could have given up work, he should have got well. Some men who followed this trade worked only on alternate days, and others at longer intervals, according to the susceptibility of the individual. The habits of Boeri were always regular, but the disease most frequently attacked the intemperate. In some, salivation was produced, but this Boeri had always the good fortune to escape.—*London Lancet.*

---

*Case of a Dwarf in whom Premature Labour was artificially induced with success.* By M. Paul Dubois.—C. L. was born in Italy, from a mother of ordinary stature, and a father only three feet and a half high, though straight and well-proportioned, who was himself the son of a dwarf, and had three dwarf sisters. He had six children, of whom three were born and remained dwarfs, and three were born well-developed, and acquired the ordinary stature.

Caroline (the subject of the present observations) presented in her early infancy nothing remarkable except her extreme diminutiveness. She was brought up with facility; she menstruated without difficulty when eleven years old, and the catamenial function has since been regularly performed, the quantity of fluid discharged being nearly the same as in a woman of ordinary size. When twelve years old she was brought to France with one of her dwarf brothers, and both were for some time publicly exhibited. At the conclusion of this engagement, when about twenty years old, she formed a connexion with a man of ordinary stature; the sexual intercourse was at first extremely painful, but she soon became pregnant. She went to the full period of gestation without any thing remarkable occurring, and on the 3d of April, 1838, the first labour-pains occurred.

At first the labour presented nothing peculiar; the pains were regular, the dilatation of the os uteri complete, the membranes burst of their own accord, and well-sustained expelling efforts continued without result till the next day. On the 5th she was attacked with convulsions, which were repeated several times, and produced first an occasional and then a permanent state of stupor. A physician was called in, but considering that the expulsion of the child could not be effected without artificial aid, he called in M. P. Dubois.

The little patient was then lying in a cradle in that sleepy state which succeeds to attacks of eclampsia, and occupies the intervals between them; there was complete abolition of sensation and intelligence, and relaxation of the limbs. The child's head was found partially engorged in the upper aperture of the pelvis, projecting into its hollow; the os uteri was completely dilated; and having ascertained that the child was dead, M. Dubois determined on applying the forceps. Notwithstanding the apparent disproportion between their size and that of the patient, they were easily though unsuccessfully applied; but without removing them, M. Dubois introduced between their blades a pointed bistoury, with which he perforated the child's head. The greater part of the brain was at once evacuated, and the head passed on to the vulva; but the small size of that aperture rendered its rupture inevitable, and it tore obliquely towards the right buttock round the anus, but leaving the sphincter uninjured.

The child without its brain weighed five pounds and a half, and was seventeen Paris inches and a half long. The mother remained in the same sleepy state till the next day, and then on recovery had no recollection of anything that had passed; the delivery was followed by some inflammatory symptoms, but they yielded to appropriate treatment.

Before leaving her, M. Dubois ascertained that the antero-posterior diameter of the upper part of the pelvis was only nearly three inches; he therefore told her her danger, and advised her if she again became pregnant to come to him very early. In September, 1839, she wrote to him that she was again pregnant, and with some difficulty the date of her conception was fixed at the early part of June; she was carefully watched during the following months, and on the 13th of February, 1840, the uterus having in the few preceding days increased considerably in size, M. Dubois determined to excite the contractions of the uterus.

At half-past 9 in the morning, a common speculum being passed into the vagina, a piece of prepared sponge, about an inch and a half long, and cone-shaped, with a base from six to eight lines in diameter, was introduced by means of a long pair of forceps into the os uteri. It was fixed in its place by a second larger piece, and threads were adapted to both so that they might be drawn out as soon as it was necessary. The patient was scarcely placed in her bed again before she felt some slight pains; forty grains of ergot of rye were almost directly after administered in doses of five grains every ten minutes; and during its administration the pains became gradually stronger and longer, and from eleven to twelve o'clock followed each other very rapidly, and were well-sustained.

At half past twelve the sponges were withdrawn, and that in the neck of the uterus was found of four times its previous size. The orifice would easily have admitted the tips of two fingers, but as it was rather rigid, some extract of belladonna was applied to it. The pains continued increasing; and at half past 1 the membranes could be reached, though rendered too tense by the great quantity of liquor amnii to permit the mode of presentation to be detected. The pulse was regular, at ninety-two. From half past 2 to half past 3 the pains diminished; they then again returned, but at long intervals, during which the patient fell into a state of profound sleep. At half past 4 she was bled to four ounces, and the pains soon became stronger. At half past 6 she had nausea, and vomited some mucus containing a little ergot of rye. From that time the pains ceased, and the patient slept calmly till 8 in the evening, when a few slight uterine contractions again took place.

The dilatation of the os uteri was scarcely altered, and the cervix retained nearly the same length up to 8 o'clock. The membranes were then ruptured, and a great quantity of fluid evacuated; the fœtus approached the orifice of the uterus, and the buttocks were found presenting. From this time the pains were regular; at 11 the cervix was almost completely obliterated, and at midnight its dilatation was nearly perfect. A 1 o'clock the pains were less vigorous and frequent, and forty grains (five every ten minutes) of ergot of rye were administered, but were nearly rejected by vomiting. At 3 the same quantity was administered in an enema; the uterine contractions then returned with full force, and by 7 in the morning of the 14th the buttocks presented at the vulva. M. Dubois now hooking his forefinger round one of the haunches, extracted the trunk without difficulty, and then disengaged the arms. The head was for some time arrested at the upper strait of the pelvis; tolerably powerful traction, during which the infant inspired several times, was necessary; and at last the head passed through, and the child was in another instant born.

It was a little girl, which was at first weak, but very quickly recovered. The expulsion of the placenta was followed by rather considerable hæmorrhage, but it was checked by friction. The mother's recovery was rapid and natural.

The infant at birth weighed three pounds and three quarters; its total length was fifteen inches; from the vertex to the umbilicus it was eight inches and one-sixth; the occipito-frontal diameter of the head was three inches and a half; the occipito-mental was four inches and a quarter; the bi-parietal three inches; the sub-occipito-bregmatic three inches and one-eighth. The mother was three feet three inches high, and on the whole well proportioned.—*Brit. and For. Med. Rev., Bulletin de l'Académie Royale de Médecine.* Mars 31, 1840.

---

*Inoculation with the Matter of "Grease" in the Horse, producing symptoms of Vaccine in he Human Subject.*—Dr. Stokes presented two drawings of the appearances in a case which had recently occurred in Dublin, which was of importance, as tending to corroborate the opinions of Jenner with respect to the origin of cowpock. For the opportunity of witnessing this case, Dr. Stokes was indebted to Mr. Pakenham, under whose care the patient had been placed. After quoting some passages from the works of Jenner, Dr. Stokes observed, that some had misunderstood Jenner's opinions on this subject, and believed that he had held that the direct inoculation with the matter of grease was capable of producing a disease the same as vaccine in man. This was not Jenner's doctrine. He says that the fluid of grease seems capable of generating a like vaccine, *after it has passed through the system of the cow.* But in speaking of the form of disease produced in man by inoculation with the matter of grease, Dr. Jenner was not distinct or accurate in his description. He speaks of ulcerated sores on the hands, inflamed lymphatics, and abscesses of the axilla, and says that many medical friends of his were aware of the similarity between the eruption on the hands after infection with grease, and that which succeeds cowpock, but he does not give any precise description of the appearances which result from inoculation with equine matter in the human subject. He states, however, that persons who have had sores on the hands from inoculation with grease, do not appear to be susceptible of small-pox, and alludes to the great difficulty frequently experienced in producing disease with variolous matters in farriers and persons who have been much engaged about horses. Dr. Stokes proceeded to read some notes of a case of equine infection, which occurred in 1793, and is detailed in Dr. Jenner's work. Three men, on receiving the infection of grease, got sores on the hands, with pains in the axillary glands, shivering, and lassitude; and two of them, who had previously gone through the small-pox, said that their sensations were similar to those they had experienced on the invasion of that malady. The whole duration of the febrile symptoms in these cases was about twenty-four hours. Dr. Stokes next exhibited a drawing of the pustule produced in a child by inoculation with matter taken from one of the men infected with grease. He also exhibited a drawing of the true vaccine pustule, and contrasted it with the former. The only apparent difference between them was, that there was a greater degree of lividity about the equine than the vaccine pustule. As a further proof of the close connection between the two poisons, Dr. Jenner states that he has never been able to discover any instance of the prevalence of the vaccine pustule among cows, which could not be traced to cows originally infected, or which had been milked by persons labouring under equine infection. The opinions put forward by Dr. Jenner on this subject were controverted by some of his contemporaries, among whom the principal was Dr. Woodville, who stated that he had made several experiments to try whether cowpock could be produced by grease, but had always failed, and that his friend, Mr. Coleman, of the Veterinary College, had made several experiments of the same kind with a similar result.

The case which had occurred to Mr. Pakenham was this: The servant of a gentleman residing near town, a man of good constitution and temperate habits, was in the daily habit of cleaning the hoofs of a horse labouring under grease. On one occasion the animal became restive; overturned the bucket in which the diseased limb was being washed, the edge of which cut the man over the upper lip. The groom immediately took up a sponge he had been using, and which was saturated with the matter of grease, and wiped his lip with it. He did the same the next day, and the day after, so that the matter was applied to the broken surface three, and perhaps four times. On the sixth day he became ill, complained of headach, lassitude, and loss of appetite. On the same evening a vesicle appeared on the upper lip, and next day another on the superior part of the cheek over the malar eminence; a third was placed more internally under the lower eyelid. Dr. Stokes saw him on the ninth day, and the appearances presented by the vesicles were such as were represented in the drawing he was about to exhibit. The drawing was taken on the tenth day. Dr. Stokes pointed out one of the pustules, and observed that on the ninth day it presented an appearance precisely similar to that of a vaccine vesicle, the areola being beutifully marked, and the vesicle so like that of a cowpock, that no distinction could be perceived. Around this vesicle there were several smaller and less regular ones. The original wound presented the appearance of a superficial eschar, and the cheek was swollen, but the constitutional symptoms were so slight, that the man was up and walking about. The case was seen by several medical men familiar with the phenomena of cowpock, and all agreed that nothing could be more like it. Dr. Stokes exhibited also another drawing of the parts taken on the fifteenth day, and observed that the appearance of the scab and of the retreating areola were very similar to those observed in the same stage of ordinary vaccinia. The chief interest of the case was, that it exhibited a form of disease originating in equine infection, and having certainly no connection with glanders. He had hoped to be able to procure a drawing of the horse's heel, but had not been so fortunate as to obtain it. The case shewed that a disease remarkably similar to vaccinia might be produced in the human subject by the matter of grease. The only points of apparent difference between them were, that in the latter the matter contained in the vesicle seemed more purulent, and the surrounding areola somewhat more livid.—*Dublin Journal of Medical Science.*

---

*On a Peculiar Monstrosity.* By M. Faesebeck, of Brunswick.—In a letter to Professor Müller on the arrangement of the cervical portion of the sympathetic nerve, the author mentions the case of a male child, twelve weeks old, and still living in his town, with a very singular abnormal formation. Above the umbilicus it is well made; but just to the left and a little above the level of that part there grows out of it a semitendinous cylindrical body, about an inch and a half thick and an inch long, on which there is a second pelvis with two extremities and sexual organs. These parts are placed transversely across the child's abdomen. The remarkable circumstance is that the child makes water through both penises; but that which flows from the second penis is only a turbid fluid like urine, and sometimes white and milky; and this last character is observed only after the child has been sucking.—*Müller's Archiv.* 1840. Heft i.

